# Isolation and Biochemical Characterization of a New Thrombin-Like Serine Protease from *Bothrops pirajai* Snake Venom

**DOI:** 10.1155/2014/595186

**Published:** 2014-02-26

**Authors:** Kayena D. Zaqueo, Anderson M. Kayano, Rodrigo Simões-Silva, Leandro S. Moreira-Dill, Carla F. C. Fernandes, André L. Fuly, Vinícius G. Maltarollo, Kathia M. Honório, Saulo L. da Silva, Gerardo Acosta, Maria Antonia O. Caballol, Eliandre de Oliveira, Fernando Albericio, Leonardo A. Calderon, Andreimar M. Soares, Rodrigo G. Stábeli

**Affiliations:** ^1^Centro de Estudos de Biomoléculas Aplicadas à Saúde, CEBio, Fundação Oswaldo Cruz, Fiocruz Rondônia e Departamento de Medicina, Universidade Federal de Rondônia, UNIR, Rua da Beira 7176, Bairro Lagoa, 76812-245 Porto Velho, RO, Brazil; ^2^Departmento de Biologia Celular e Molecular, Instituto de Biologia, Universidade Federal Fluminense, 24210-130 Niteroi, RJ, Brazil; ^3^Centro de Ciências Naturais e Humanas, Universidade Federal do ABC, 09210-170 Santo André, SP, Brazil; ^4^Escola de Artes, Ciências e Humanidades, USP, 03828-000 São Paulo, SP, Brazil; ^5^Universidade Federal de São João Del Rei, UFSJ, Campus Alto Paraopeba, 36420-000 Ouro Branco, MG, Brazil; ^6^Institute for Research in Biomedicine (IRB Barcelona), 08028 Barcelona, Spain; ^7^CIBER-BBN, Barcelona Science Park, 08028 Barcelona, Spain; ^8^Proteomic Platform, Barcelona Science Park, 08028 Barcelona, Spain; ^9^Department of Organic Chemistry, University of Barcelona, 08028 Barcelona, Spain; ^10^School of Chemistry, University of KwaZulu Natal, Durban 4001, South Africa

## Abstract

This paper presents a novel serine protease (SP) isolated from *Bothrops pirajai*, a venomous snake found solely in Brazil that belongs to the Viperidae family. The identified SP, named BpirSP-39, was isolated by three chromatographic steps (size exclusion, bioaffinity, and reverse phase chromatographies). The molecular mass of BpirSP-39 was estimated by SDS-PAGE and confirmed by mass spectrometry (39,408.32 Da). The protein was able to form fibrin networks, which was not observed in the presence of serine protease inhibitors, such as phenylmethylsulfonyl fluoride (PMSF). Furthermore, BpirSP-39 presented considerable thermal stability and was apparently able to activate factor XIII of the blood coagulation cascade, unlike most serine proteases. BpirSP-39 was capable of hydrolyzing different chromogenic substrates tested (S-2222, S-2302, and S-2238) while Cu^2+^ significantly diminished BspirSP-39 activity on the three tested substrates. The enzyme promoted platelet aggregation and also exhibited fibrinogenolytic, fibrinolytic, gelatinolytic, and amidolytic activities. The multiple alignment showed high sequence similarity to other thrombin-like enzymes from snake venoms. These results allow us to conclude that a new SP was isolated from *Bothrops pirajai* snake venom.

## 1. Introduction

Snake venoms have proteolytic enzymes that can be divided into two main groups: metalloproteases and serine proteases, which affect the hemostatic system through different mechanisms [1, 2]. Serine proteases are abundant in snake venoms, particularly of the Viperidae family, and may constitute up to 20% of total venom proteins. This class of enzymes has a highly conserved catalytic triad (His57, Asp102, and Ser195) [3]. Besides venomous snakes, these enzymes are often found in many organisms such as viruses, bacteria, and higher mammals. Serine proteases can participate in several biological activities, such as complementing system activation, cell differentiation and homeostasis and even prey digestion [4–7]. This class of proteases affects different steps of the coagulation cascade, often nonspecifically, by proteolytic degradation. Selectively, they can activate or inactivate specific coagulation factors involved in platelet aggregation, coagulation, and fibrinolysis [8].

In snake venoms, one class of serine proteases, named snake venom thrombin-like enzymes (svTLEs), possesses coagulant activity similar to human thrombin. They convert fibrinogen to fibrin by the cleavage of the A*α* and B*β* chains [2]. Some of these enzymes are able to cleave only the *α* or *β* chains or both chains of the fibrinogen and are therefore known as svTLE-A, svTLE-B, or svTLE-AB, respectively [9]. New serine proteases are constantly being described and/or characterized [10–13].

The aim of the present study is the isolation and biochemical characterization of a new serineprotease from *Bothrops pirajai* snake venom. Because it is such a rare snake and lives in a small area of the world, little about its venom has been described to date; these studies are limited to the purification of (i) phospholipase A_2_: piratoxin-I [14], piratoxin-II and -III [15], MP-III 4R [16], and BpirPLA_2_-I [17], (ii) C-type lectin: BPL [18], (iii) LAAO: BpirLAAO-I [19] and (iv) two serine proteases: BpirSP27 and BpirSP41 [20].

## 2. Materials and Methods

### 2.1. Isolation and Molecular Mass Determination

The identified serineprotease (BpirSP-39) was isolated after chromatographic fractionation of *B. pirajai* venom by size exclusion, followed by bioaffinity and reverse phase chromatographies. So, about 40 mg of crude venom was solubilized in 1 mL of 20 mM Tris-HCl pH 7.6 and centrifuged at 9000 ×g for 10 min at room temperature. The clear supernatant was applied to a Superdex G-75 (70 × 0.9 cm) column (GE Healthcare), preequilibrated with 20 mM Tris-HCl pH 7.6, and the chromatography was carried out at a flow of 0.75 mL/min, collecting fractions of 1 mL/tube. The elution of proteins was monitored at 280 nm.

Fractions with coagulant activity were lyophilized, suspended in 50 mM Tris-HCl pH 7.4 plus 0.5 M NaCl, and applied to a Hitrap benzamidine *Fast Flow* column (GE Healthcare), previously equilibrated with 50 mM Tris-HCl pH 7.4 plus 0.5 M NaCl. The elution of proteins was performed using 0.5 M NaCl plus 10 mM HCl at a flow of 1 mL/min. The collected samples (1 mL) were desalted and lyophilized.

The fraction of interest was dissolved in 0.1% trifluoroacetic acid (TFA) and a reversed-phase high-performance chromatography was performed using a C2/C18 column (10 mm × 4.6 mm, 3 *μ*m, 120 Å) (GE Healthcare), preequilibrated with 0.1% TFA. The elution was carried out using a linear gradient of 0–100% (99% acetonitrile plus 0.1% TFA) at a flow rate of 0.75 mL/min. All chromatographic steps were performed in an Akta Purifier 10 system (GE Healthcare).

The molecular mass was estimated by 12.5% SDS-PAGE [21] and determined by mass spectrometry in an AXIMA TOF^2^ system. The mass spectrum was acquired in linear mode, using a saturated solution of sinapinic acid as ionization matrix.

### 2.2. Enzyme Activities

#### 2.2.1. Determination of Coagulant Activity

The minimum coagulant dose (MCD), or the amount of enzyme capable of coagulating 200 *μ*L of plasma in 60 sec, was determined visually using different concentrations of isolated protein (0.5–3.0 *μ*g) and citrated human plasma [22]. The time needed to form fibrin networks was measured by a chronometer and the results were expressed in percentage of seconds (1/Δ  × 100), where Δ is the average time in seconds. The action of protease inhibitors on the purified enzyme was evaluated determining coagulation activity after the incubation of 2 *μ*g serineprotease with heparin, citrate, ethylenediaminetetraacetic acid (EDTA), and phenylmethanesulfonyl fluoride (PMSF) for 20 minutes at room temperature. The thermal stability of the protease was verified by measuring coagulation activity after preincubation of 2 *μ*g protein at different temperatures (−70°C–85°C) for 30 minutes. The assays were carried out in duplicate with *n* = 3.

#### 2.2.2. Activation of Factor XIII of the Clotting Cascade

After centrifugation of heparinized blood samples at 2205 ×g for 15 minutes, 400 *μ*L plasma was incubated with (i) 2 *μ*g BpirSP-39 (40 *μ*L), or (ii) 2 *μ*g BjussuSP-I (a serineprotease from *B. jararacussu* that is not able to activate factor XIII), or (iii) 40 *μ*L of water, dilution the sample solution (negative controls) and (iv) blood collected without anticoagulant (positive control) in order to evaluate the stability of the formed fibrin network [23]. Aiming at evaluating the activation of coagulation factor XIII, 200 *μ*L of 10 M urea solution was added to the clots and the samples were incubated for 48 h at 37°C. The assays were carried out in duplicate with *n* = 3.

#### 2.2.3. Activity on Synthetic Substrates

The ability of SP in hydrolyzing chromogenic substrates (0.1 mM, final concentration) S-2238 (that is suitable for thrombin-like enzymes), S-2222 (for factor Xa) and S-2302 (for plasma kallikrein, factor XIa and XIIa), was analyzed using a Thermomax microplate reader (Molecular Devices, Menlo Park, CA, USA). The enzymatic reaction was monitored for 20 min. at 37°C and A405 nm. The effective concentration (EC) was determined as the concentration of SP (*μ*g/mL) able to produce an increase of 0.3/5 minutes. SP was pre-incubated either with EDTA (20 mM), PMSF (2 mM), benzamidine (15 mM), and O-Phe (0.3 mM) for 60 min at 37°C or with 10 mM divalent cations (Cu^2+^, Mn^2+^, Ba^2+^, and Ca^2+^) for 30 min at 37°C, and then the reaction was started by adding substrates [24, 25].

#### 2.2.4. Platelet Aggregation Assays

Washed rabbit platelets (WRP) were prepared according to the procedure described by Fuly and coworkers [26]. Collected blood plus 5 mM EDTA was centrifuged at 360 ×g for 12 min at room temperature, and the Platelet Rich Plasma (PRP) obtained was further centrifuged at 1370 ×g for 20 minutes. The platelet pellets were suspended in a calcium-free Tyrode's solution containing 0.35% (w/v) bovine serum albumin (BSA) plus 0.1 mM EGTA (final concentration) pH 6.5 and washed twice by centrifugation. The final pellet was then suspended in Tyrode-BSA pH 7.5, without EGTA. The suspension was adjusted to 3-4 × 10^5^ platelets/mL and platelet aggregation was measured by turbidimetry using a dual Whole Blood Lumi-Aggregometer (model 490 2D, Chrono-Log Corporation). The assays were performed at 37°C in siliconized glass cells using 200 *μ*L of WRP, under stirring conditions, and aggregation was triggered after preincubation for 2 min with aliquots of SP in the presence of 1.0 mM CaCl_2_ (final concentration).

#### 2.2.5. Fibrinogenolytic Activity

The fibrinogenolytic activity of SP was determined according to Cominetti and coworkers [27] with modifications. Samples of bovine fibrinogen (10 mg/mL) were incubated with different concentrations of enzyme (0.5–3 *μ*g) at 37°C for 2 hours. The reactions were stopped by adding 0.5 mM Tris-HCl pH 8.0, 20% glycerol (v/v), 4% SDS (v/v), 0.05% bromophenol blue (w/v), and 0.3% DL-dithiothreitol (w/v), in a 1 : 1 proportion. After overnight incubation, the digested fibrinogen was analyzed using 10% SDS-PAGE.

#### 2.2.6. Fibrinolytic Activity

Fibrinolytic activity was evaluated according to the method described by Cominetti and coworkers [27] and Chudzinski-Tavassi and Modesto [23]. First enough agarose to prepare a 0.9% gel was solubilized in 50 mM Tris-HCl pH 7.4 plus 100 mM CaCl_2_ and heated until melted. At a temperature of 37°C, 0.3% bovine fibrinogen, dissolved in 50 mM Tris-HCl pH 7.4 plus 100 mM CaCl_2_, and 1.2 U/mL bovine thrombin were added to the agarose solution. Afterwards, the mixture was polymerized in a Petri dish (0.9 cm × 15 cm) and the SP (5 and 10 *μ*g in PBS), crude venom (3 *μ*g, positive control), and PBS (negative control) were incubated at 37°C overnight in orifices as previously done. The halos that formed indicating fibrinolytic activity were analyzed by comparison to negative and positive controls. An activity unit was defined as the quantity of protein capable of producing a 1 mm halo on fibrin gel. The result was expressed in millimeters.

#### 2.2.7. Gelatinolytic Activity Assay

Gelatinolytic activity was assessed according to the procedure described by Cominetti and coworkers [27]. SDS-PAGE was carried out on 12.5% gel containing 0.3% gelatin as a copolymerized substrate under nonreducing conditions [21]. After electrophoresis, the gel was washed twice in 0.5% Triton X-100 (v/v) for 30 min to remove SDS and incubated in 50 mM Tris-HCl pH 8.0 plus 5 mM CaCl_2_ at 37°C for 20 h. Then, the gel was stained with Coomassie blue R-250 and gelatinolytic activity was observed by the presence of clear proteolytic zones.

#### 2.2.8. Amidolytic Activity on Substrate BA*p*NA

Amidolytic activity was measured after incubation at 37°C for 5 h of 10 *μ*g BpirSP-39 in 500 *μ*L solution containing 1% N^*α*^-Benzoyl-DL-Arginyl *p*-nitroanilide (BA*p*NA) in 100 mM Tris-HCl pH 8.0. The reaction's product was analyzed at 405 nm using a value of 8800 M^−1^·cm^−1^ as the molar extinction coefficient of p-nitroanilide. The negative control was carried out using water plus BA*p*NA. A unit of enzymatic activity was defined as the quantity of enzyme capable of releasing 1 *μ*mol p-nitroanilide/min, corresponding to the increase of 0.009 absorbance units measured at A405 nm.

### 2.3. Sequencing Determination

#### 2.3.1. Solution Digestion

The protein was reduced by treatment with a solution of 20 mM DTT in 50 mM NH_4_HCO_3_ for 1 h at 30°C and alkylated with a solution of 150 mM iodine acetamide in 50 mM NH_4_HCO_3_ for 1 h at 30°C. The sample was then digested overnight at 37°C with trypsin (sequencing grade modified, Promega). Afterwards, tryptic peptides were cleaned up with a Proxeon Stage tip and eluted with 70% acetonitrile/0.1% trifluoroacetic acid. The eluted peptides were dried in a vacuum centrifuge and resuspended in 1% formic acid for LC-MS/MS analysis. Mass spectrometry was performed in a nanoAcquity (Waters) HPLC coupled to an Orbitrap Velos mass spectrometer (Thermo Scientific). An aliquot of the tryptic digest was injected and separated in a C18 reverse phase column (75 *μ*mOi, 10 cm, nanoAcquity, 1.7 *μ*m BEH column, Waters). Bound peptides were eluted with the following gradients: 1 to 40% B in 20 minutes, followed by 40 to 60% B in 5 min; flow was 250 nL/min (A: 0.1% formic acid in water; B: 0.1% formic acid in acetonitrile). Eluted peptides were ionized in an emitter needle (PicoTipTM, New Objective). Spray voltage applied was 1900 V. Peptide masses (*m/z*: 300–1700) were measured in full scan in the Orbitrap at a resolution of 60,000 at 400 *m/z*. Up to the 5 most abundant peptides (minimum intensity of 1500 counts) were selected from each MS scan and fragmented in the HCD collision cell using a normalized collision energy (NCE) of 40% with nitrogen as the collision gas. Fragments were detected in the Orbitrap with a resolution of 7500 FWHM at 400 *m/z*. Raw data were collected using Thermo Xcalibur (v.2.1.0.1140).

#### 2.3.2. Database Search

Raw data were analyzed using Proteome Discoverer (v.1.3.0.339) software. A search was run with the search engine MASCOT against the NCBInr Serpentes database. Also, an  .mgf file was generated in Proteome Discoverer and this file was used to search with PEAKS Studio (v.5.3.) against the same database. After that the homology search tool SPIDER* was used to run a tag homology search. The search parameters were Database/Taxonomy: NCBInr Serpentes; missed cleavage: 2; fixed modifications: carbamidomethyl of cysteine; variable modifications: oxidation of methionine and pyro-Glu (N-term Glutamine); peptide tolerance: 10 ppm for MS spectra and 0.05 Da for MS/MS spectra; and enzyme: trypsin.

The Percolator node was used in the Proteome Discoverer Mascot search in order to discriminate correct from incorrect peptide spectrum matches using the *q*-value (FDR) to improve the number of confidently identified peptides at a given false discovery rate. The results have been filtered so only high confidence peptides (FDR ≤ 0.01) are considered for identification results.

### 2.4. BpirSP-39 Molecular Modeling and Determination of N-Glycosylation Sites

The structural model of the BpirSP-39 from *Bothrops pirajai* was generated employing the threading modeling method [28–30], which was performed using the HHpred software [31] available at http://toolkit.tuebingen.mpg.de/hhpred. Initially, HHpred generated 112 alignments for BpirSP-39. The alignments were obtained using the global mode and the gaps resulting from LC-MS/MS sequencing were filled by homology with a thrombin-like enzyme from *Agkistrodon halys *venom (PDB ID: 4E7N) [32] (selected to construct the model of BpirSP-39). The first two gaps were confirmed by Edman's degradation (data not showed), and the third and fourth gaps are justified by the large amount of lysine which generated small fragments not detected by LC-MS/MS. The chosen template showed the best alignment score (286.73) and the identity between the studied protein sequence and the template was 67%. Potential N-glycosylation sites of serineprotease were predicted employing NetNGlyc v.1.0, [33] available at http://www.cbs.dtu.dk/.

#### 2.4.1. Simulation of Molecular Dynamics

After the construction of the initial model, we performed simulations of molecular dynamics (MD) of the studied protein. All the MD parameters were equally set to the two generated models. The MD simulations were performed employing GROMACS (GROningenMAchine for Chemical Simulation) v.4.5.4 software [34, 35] in Intel Xeon processor with 8 GB RAM, operating in a CentOS 5.5 Linux operational system. The simple point charge (SPC) model was used to represent explicit water molecules. Protonation states of charged groups were set according to pH 7.0 and counter ions were added to neutralize the system. GROMOS force field [36] was chosen to perform the MD simulation. These simulations were performed at constant temperature and pressure in a periodic truncated cubic box, and the minimum distance between any atom of the protein and the box wall was 1.0 nm.

Initially, an energy minimization using the steepest descent algorithm was performed. After that 20 ps of MD simulation with position restraints applied to the protein was performed at 298 K to relax the system. And finally, an unrestrained MD simulation was performed at 298 K for 10 ns to assess the stability of the structures. During the simulation, temperature and pressure (1.0 bar) were maintained by an external bath controlling heat and isotropic pressure.

#### 2.4.2. Structural Analysis and Validation

The model generated after the MD simulation was checked using several GROMACS structural analyses, as well as the analysis of Ramachandran plot generated with Rampage [37]. The pseudo-energy profile of the models was analyzed with Verify 3D [38, 39] available at<http://nihserver.mbi.ucla.edu/Verify_3D/>and ProSA-web [40, 41].

## 3. Results 

**Figure 1:**
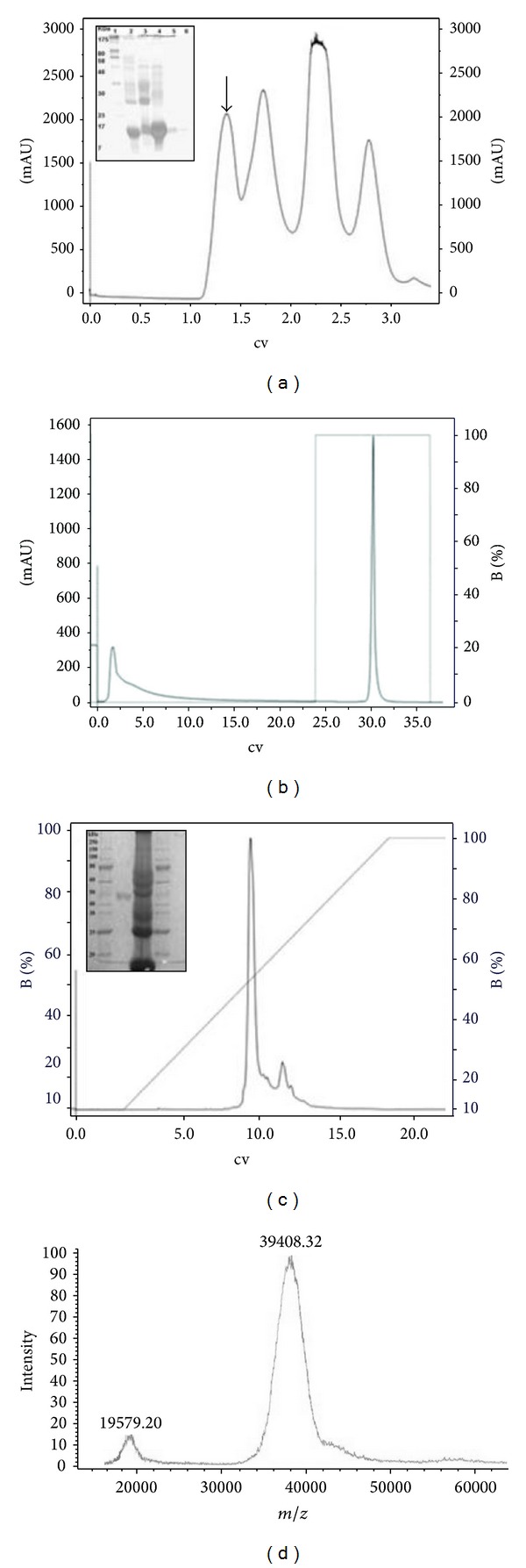
Purification profile of the serineprotease BpirSP-39 from *Bothrops pirajai* crude venom. The detached arrow (a) indicates the fraction with the highest coagulation activity, fraction 1 of 12.5% SDS-PAGE in denaturing conditions. Line 1: molecular mass standard, Color Plus Prestained Protein Marker, Broad Range (7–175 kDa) (P7709S New England Biolabs), lines 2–6: Fractions 1–5 obtained after chromatography. (b) Affinity chromatography of fraction 1 on benzamidine sepharose column. (c) High performance liquid chromatography using the C2/C18 column (10 mm × 4.6 mm, 3 *μ*m, 120 Å) and 12.5% SDS-PAGE of BpirSP-39 and *B. pirajai* crude venom. Lines 1 and 4: molecular mass standard: Protein Ladder (10–250 kDa) (P7703S New England Biolabs); 2- BpirSP-39 in denaturing conditions showing a band of approximately 49 kDa; 3-crude venom of *B. pirajai *in denaturing conditions. (d) Mass spectrum of BpirSP-39 determined by AXIMA TOF^2^. The identified protein presented a molecular mass of 39,408.32 Da. The peak at 19,579.02 Da indicates the double charge of the protein. The absorbance was monitored at A280 nm.

The purification of BpirSP-39 was performed using three consecutive chromatographic steps. The first step of the *B. pirajai* venom fractionation, performed by size-exclusion molecular chromatography on Superdex G-75, resulted in five fractions (P1–P5) (Figure 1(a)). The peaks P-1 and P-2 were capable of coagulating the citrated plasma and promoting proteolytic activity, when the chromogenic substrate BA*p*NA was used. Since P-1 demonstrated the highest coagulant and proteolytic activities, it was fractioned by affinity chromatography using a benzamidine Sepharose column resulting in two peaks (Figure 1(b)). The coagulant fraction was applied to a C2/C18 column, and after elution enzymatic activity was observed in the first fraction (Figure 1(c)).

The relative molecular mass of SP, estimated by SDS-PAGE 12.5%, was approximately 49 kDa (Figure 1(a)) but when determined by mass spectrometry it was 39.408,32 Da (Figure 1(d)). Knowing that mass spectrometry is a more accurate method than polyacrylamide electrophoresis, the new identified serineprotease was called BpirSP-39.

**Figure 2:**
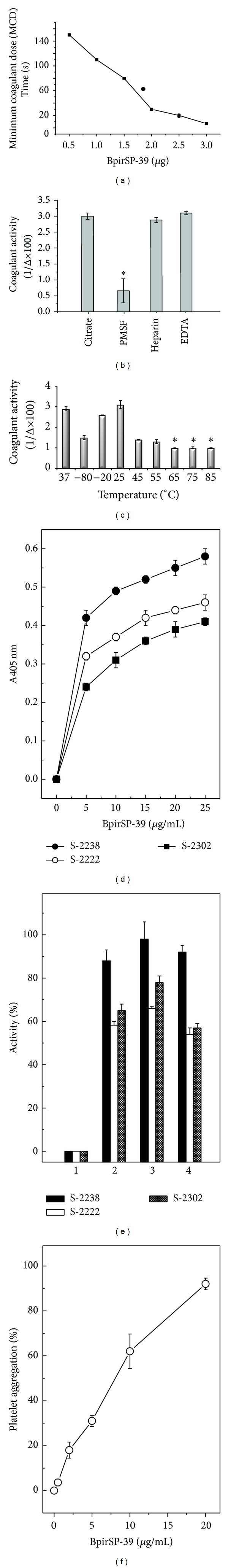
Enzymatic characterization of the protein. (a) Determination of minimum coagulant dose (MCD) of BpirSP-39 using a concentration-response curve. The time of formation of fibrin network was measured by chronometer and the samples were evaluated visually. (b) Evaluation of inhibitors' action on the coagulation activity of BpirSP-39. (c) Evaluation of BpirSP-39 thermostability on the clotting of human citrated plasma. (d) Effect of protein on different chromogenic substrates. (e) Effect of metals on the enzymatic activity of serineprotease (column 1, Cu^2+^; column 2, Mn^2+^; column 3, Ba^2+^; and column 4, Ca^2+^). (f) Effect of serineprotease on platelet aggregation. Results are expressed as means ± SD of two individual experiments (*n* = 3).

BpirSP-39 is a serineprotease that presents coagulant activity in citrated plasma in a concentration-dependent manner, with a minimum coagulant dose (MCD) determined to be 1.7 *μ*g of the protein (Figure 2(a)).

In contrast to the majority of snake venom serine proteases [42], BpirSP-39 is apparently able to activate the clotting cascade factor XIII and, as observed in the positive control, the fibrin network showed stability after 48 h incubation. The clot induced by BjussuSP-I was dissolved in less than 120 seconds which indicates that factor XIII was not activated. The second negative control (40 *μ*L of water) was not able to induce a coagulation process, proving that thrombin was neutralized by heparin and does not participate in the coagulation induced by BpirSP-39, though for a definite conclusion, it needs to be tested with purified factor XIII.

BpirSP-39 clotting activity was not influenced by different thrombin inhibitors (citrate, heparin, and EDTA), which distinguishes it from most svTLEs (see Table 1). However, BpirSP-39 clotting activity was significantly reduced after incubation with PMSF (Figure 2(b)). BpirSP-39 also proved to be a thermo-stable enzyme (Figure 2(c)), exhibiting highest activity at room temperature (25°C).

The enzyme possesses high catalytic activity on different chromogenic substrates tested (S-2238, S-2222, and S-2302) (Figure 2(d)); however, when incubated with Cu^2+^, its catalytic activity was diminished significantly on the three tested substrates. While Mn^2+^ influenced the activity on substrates S-2222 (for factor Xa) and S-2302 (for plasma kallikrein, factor XIa and XIIa), Ba^2+^ and Ca^2+^ had no influence on the catalytic activity on substrate S-2238 (that is suitable for thrombin-like enzymes) but modified the enzyme's activity on substrates S-2222 and S-2302. The protein was also capable of promoting platelet aggregation in a concentration-dependent manner (Figure 2(f)).

**Figure 3:**
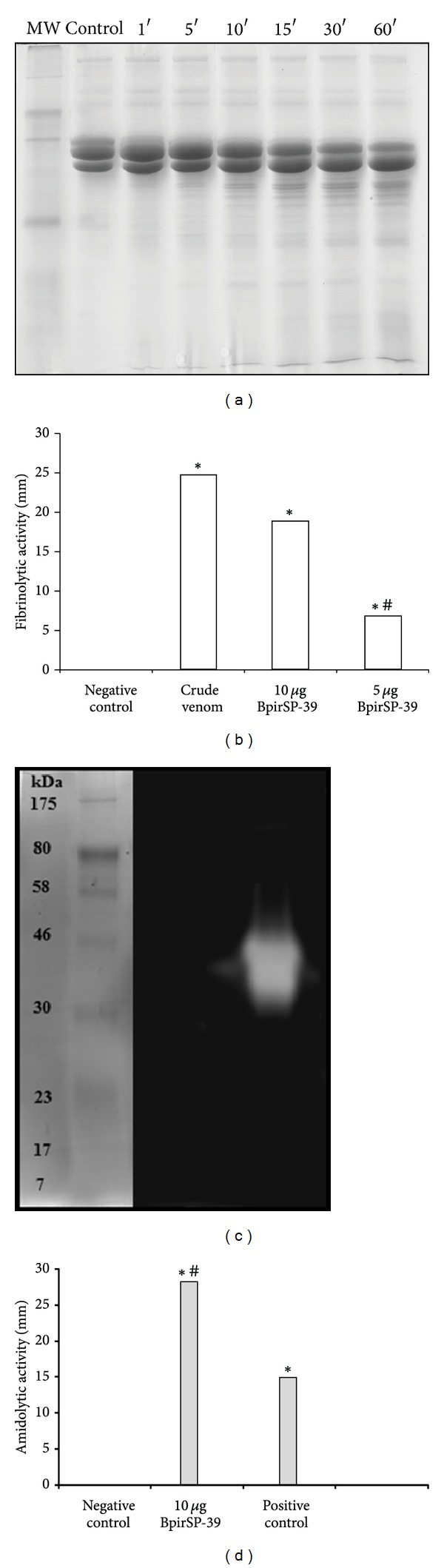
Enzymatic characterization of BpirSP-39. (a) Fibrinogenolytic activity demonstrating the degradation of the *α* and *β* chains of bovine fibrinogen. (b) Fibrinolytic activity. The data are expressed in millimeters. (c) Gelatinase activity stained with Coomassie R-250. (d) Amidolytic activity on chromogenic substrate BA*p*NA. The crude venom of *B. pirajai* was used as positive control and the dilution buffer of the sample as negative control. (∗) Values significantly different from the negative control (*P* ≤ 0.05) and (*#*) values significantly different from positive control (*P* ≤ 0.05).

The proteolytic activity of BpirSP-39 on fibrin (Figure 3(b)) demonstrates that the purified serineprotease is a fibrinogenolytic enzyme similar to other svTLEs [43]. Furthermore, BpirSP-39 showed gelatinolytic activity (Figure 3(c)) and amidolytic activity on BA*p*NA (Figure 3(d)).

**Figure 4:**
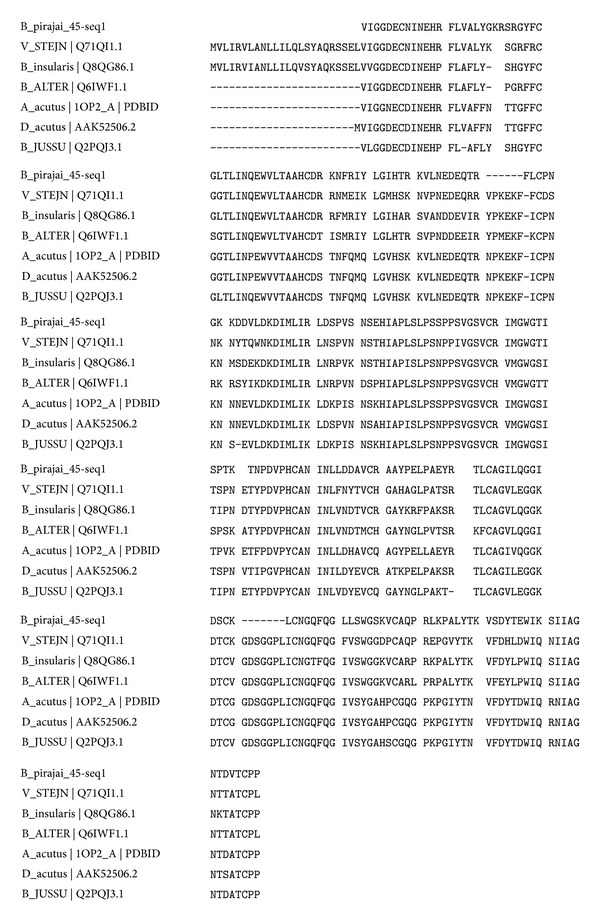
Multiple sequence alignment between BpirSP-39 and other serine proteases.

**Figure 5:**
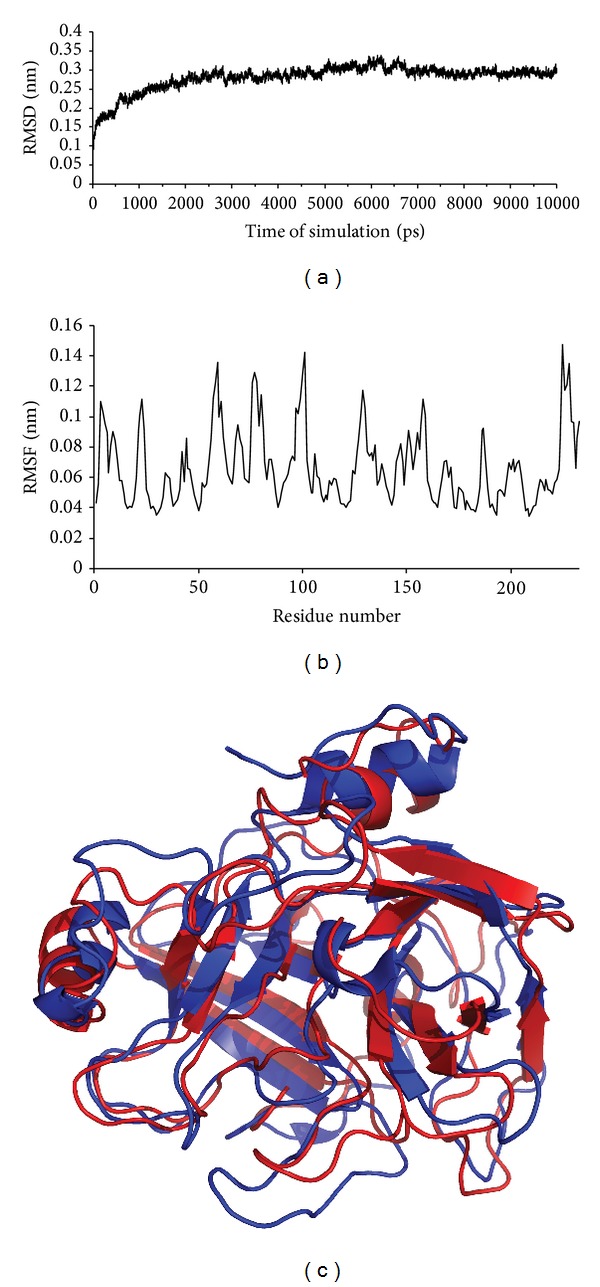
(a) RMSD versus MD simulation time for the generated model; (b) root mean squared fluctuation (RMSF) of average structure of BpirSP-39 (between 7.5 and 10 ns of MD simulation); (c) alignment between BpirSP-39 (red) model and crystallographic template (blue).

The amino acid sequence of BpirSP-39 was determined by MS/MS and showed a multiple sequence alignment between the enzyme and other serine proteases (Figure 4).  Figure 5(a) displays the root mean squared deviation (RMSD) of the backbone during the MD simulation and we can see that the structure of the BpirSP-39 model was clearly stabilized after 7500 ps. From these results, it is possible to say that the MD simulations were important to minimize the system. From the RMS fluctuation plot between 7500 and 10000 ps of MD simulation (Figure 5(b)), we can note that only the loop regions had deviation high values. The average fluctuation of the protein structure is around 0.6 Å and the maximum fluctuation is around 1.4 Å, indicating a high level of stability. These structural findings confirm the quality of the generated model.  Figure 5(c) shows the alignment between the final model and the chosen template, indicating that the predicted tertiary structure was preserved during the MD simulation.

**Figure 6:**
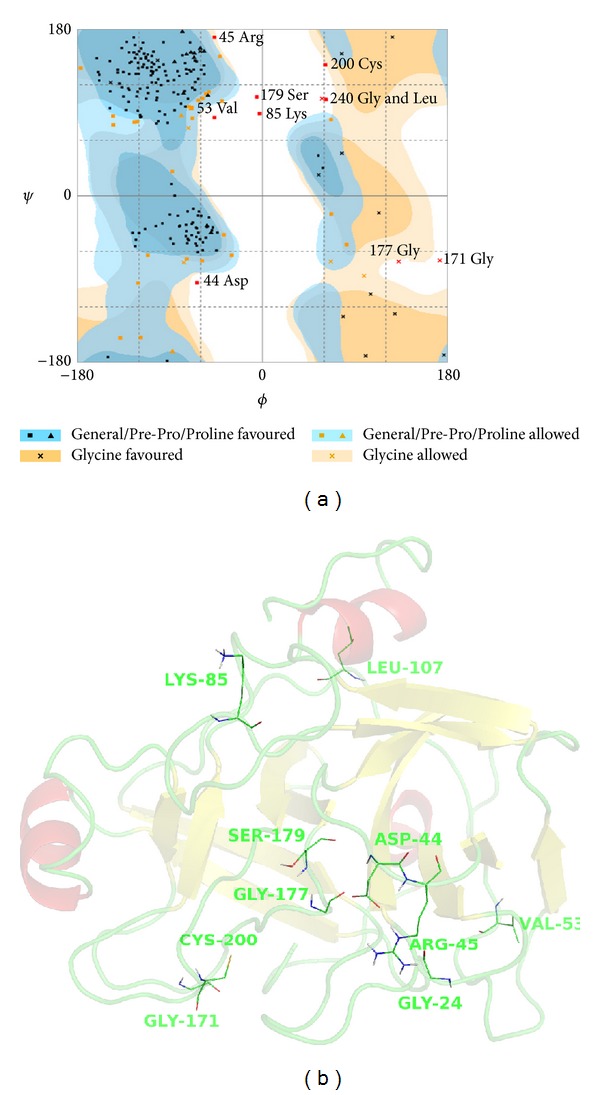
Residues located at outlier regions predicted by Ramachandran plot.

After the MD simulation, the final model presented Verify3D scores above zero for all residues, suggesting that the conformation of individual residues was adequate. Analyzing the Ramachandran plot of the final model, the BpirSP-39 structure shows 81.3% of the residues located at allowed regions and only 4.3% in outlier regions (Figure 6(a)). Also, all disordered predicted residues were located at loop regions (Figure 6(b)), suggesting that the conformation of the final model has good stereochemical quality.

**Figure 7:**
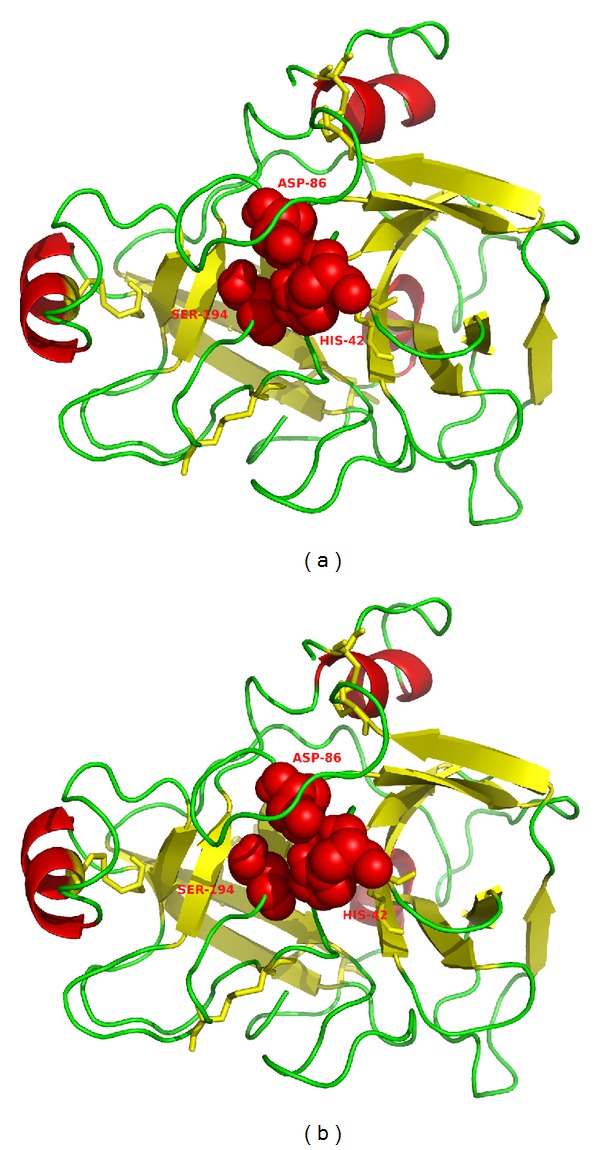
Stereoview of the final 3D model for BpirSP-39.

Finally, we validated the final model using the energy profile from ProSA web server. The initial model contained a high energy region and the final model has no region with energy higher than 0, indicating that the MD simulation was important in improving the model's quality (Supplementary Material—Figure 8, available on line at http://dx.doi.org/10.1155/2014/595186). The binding site of the modeled serineprotease is composed of a histidine, an asparagine, and a serine (His42, Asp86, and Ser194).  Figure 7 displays the obtained model with disulfide bonds in yellow and the catalytic triad in red. The final model presented the same number of disulfide bonds as other snake venom serine proteases.

Based on these results, the new identified serineprotease mimics several thrombin characteristics (Table 2).

## 4. Discussion

The present report details the isolation and biochemical characterization of BpriSP-39, a new thrombin-like enzyme from *Bothrops pirajai* snake venom, with common procedures for the isolation of snake venom serine proteases [44–50].

The divergences observed between the relative and absolute mass of BpirSP-39 were also detected with other thrombin-like proteins purified from snake venoms [51–54]. Like the majority of serine proteases [12, 13, 20, 44–48], these differences indicate that BpirSP-39 seems to be a glycosylated protein. The difference detected during electrophoretic migration was probably caused by the carbohydrate microheterogeneity of the enzyme since this fraction can vary the weight of the serine protease up to 30%. Castro and coworkers [9] suggest that the glycidyl domain can aid in structural stabilization and participate in the recognition of substrates by the enzyme.

The MCD of BpirSp-39 was 1.5 *μ*g which is similar to BjussuSP-I, a serineprotease isolated from the venom of *Bothrops jararacussu*, a venomous snake phylogenetically similar to *Bothrops pirajai *[48]. When compared to two other serineprotease isoforms isolated and characterized from the same species by Menaldo and coworkers [20] (BpirSP27 and BpirSP41, with MCDs of ~3.5 *μ*g and ~20 *μ*g, resp.), BpirSP-39 presented a higher coagulant potential. Other purified serine proteases such as PA-BJ and Jararassin-I from *Bothrops jararaca *venom show considerably reduced coagulant activity, with MCDs of 5 of 10 *μ*g, respectively [55, 56].

The new isoform of serineprotease from *Bothrops pirajai *is apparently able to activate factor III in XIIIa. It is known that factor XIII is a protransglutaminase activated by thrombin at the end of blood cascade system [57]. In plasma, factor XIII presents two subunits. While subunit A is the active form of the enzyme, subunit B plays the role of a carrier protein [58]. Factor XIIIa modifies the structure of the clot by forming cross-links between the fibrin by a link *ɛ* (*γ*-glutamyl) lysine [59] leading to increased resistance to fibrinolysis. Factor XIIIa is also able to maintain its structure when exposed to denaturing agents.

BpirSP-39 has clotting activity and its action was not influenced by different thrombin inhibitors. However, this proclotting activity was significantly reduced after incubation with PMSF, indicating that serine residues present in the enzyme's catalytic site participate in the proteolytic activity, since PMSF binds covalently to reactive serine residues present in the catalytic site [60]. Because the clotting activity was not inhibited by heparin, a direct thrombin inhibitor, it can be proposed that the identified protein acts as a thrombin-like enzyme, and not as a prothrombin activator, as is true of some snake serine proteases. If BpirSP-39 acted indirectly, activating prothrombin, the resulting thrombin would be inactivated by heparin preventing fibrin network formation. In this same way, the clotting activity of Agacutase, a recent thrombin-like enzyme isolated from *Deinagkistrodon acutus *[12], was not influenced by heparin or hirudin, which is different from BjussuSP-I, a serineprotease from *Bothrops jararacussu *whose clotting ability was reduced by heparin [48].

The BpirSP-39 showed a high thermostability similar to BpirSP27 and BpirSP41 [20], BjussuSP-I [48], and Barnettobin, a coagulant thrombin-like enzyme isolated and characterized from *Bothrops barnetti* venom [10]. This data confirms the expected results of an enzyme belonging to this class, which possesses considerable thermal stability, differing from metalloproteases which are quickly inactivated when exposed to extreme variations in temperature and pH [43].

The results obtained related to the substrate S-2238 for BpirSP-39 are similar to the data from BpirSP27 and BpirSP41 [20]. These isoforms showed reduced thrombin-like activity when incubated with Cu^2+^, BpirSP27 activity was influenced by Mn^2+^, and neither was modified when incubated with Ba^2+^ and Ca^2+^ (Figure 2(e)).

Concerning the enzyme's capacity to promote platelet aggregation, BpirSP-39 seems to be more active compared to other isolated isoforms of the same species [20]. The new isolated serineprotease was able to degrade fibrinogen and induce fibrin network formation, as well as cleave the *α* and *β* chains of bovine fibrinogen (Figure 3(a)). This is in contrast to other thrombin-like enzymes, which cleave preferentially either the *α* or *β* fibrinogen chains, occasioning an increase in fibrinopeptides A or B and consequently generating abnormal blood clots [62, 63].

As for BpirSP-39's proteolytic activity upon fibrin, gelatin, and the amidolytic substrate BA*p*NA, the purified serineprotease demonstrates enzymatic activities similar to other svTLEs [43]. It is known that the proteolytic action on BA*p*NA occurs between the amino acids Arg and Gly. This confirms that BpirSP-39 possesses fibrinogenolytic action on the *α* and *β* chains of fibrinogen, since the *in vivo* conversion of fibrinogen to fibrin carried out by thrombin is obtained by the cleavage of four peptide bonds in the amino terminal regions of the polypeptide chains 2A*α* and 2B*β*, which occur between the amino acids Arg^14^-Gly^17^ of the A*α* chains and Arg^14^-Gly^15^ of the B*β* chains [64].

The best template found for molecular modeling was the structure in the PDB registered under the number 4E7N, corresponding to a thrombin-like enzyme isolated from the venom of the snake *Agkistrodon halys*. This template has 67% identity with the sequence of *B. pirajai *and a similarity score of about 286.7. The literature says that templates with percent identities above 30% are sufficient to predict the three-dimensional structure between template-protein and target-protein.

Two potential glycosylation sites on BpirSP-39 were identified at positions Asn05 and Asn74 using the software NetNGlyc (results not shown). The presence of these sites is conserved in SVSPs. BpiSP-39 also showed the presence of twelve cysteine residues, ten of which form five disulfide bonds. The other two cysteines form a unique bridge conserved among SVSPs, in this case, involving Cys226 found in the C-terminal extension [55].

Medical and scientific interest in thrombin-like enzymes has increased considerably because of their specificity when compared to thrombin, a multifunctional enzyme [65]. These serine proteases seem to be promising defibrinogenation agents. The enzymes ancrod (Arwin), isolated from the venom of *Calloselasma rhodostoma*, and batroxobin (Defibrase), isolated from *B. moojeni*, are being used in patients suffering from thrombosis, myocardial infarction, peripheral vascular diseases, acute ischemia, and renal transplant rejection [66, 67]. Ancrod has also been used as a treatment for heparin-induced thrombocytopenia [68] without any impact on platelets [69]. Besides that, batroxobin (isolated from *Bothrops atrox*) and gyroxin, a serineprotease described by Bacila [70] and purified by Alexander and coworkers [71] from the venom of *Crotalus durissus terrificus*, are used to prepare fibrin sealants that can be utilized in different medical situations [72].

The fibrin sealants made by fibrinogen extracted from large animals and thrombin-like enzymes extracted from snake venoms were tested in both animals and humans and have diverse advantages, such as quick, easy, and cheap production; they have a large diversity of applications; they are safe, since they do not produce notable adverse reactions; and they do not use human blood or present risk of infectious disease transmission [73].

In summary, a novel isoform of serineprotease was isolated and characterized from the crude venom of the *Bothrops pirajai* snake. BpirSP-39 is a thrombin-like protein. Based on its characteristics, the enzyme could be an alternative to thrombin in the production of fibrin sealants, such as autologous fibrinogen. The enzyme, by itself, does not induce viral contamination and it also shows promising use in the treatment of clotting dysfunction.

## Supplementary Material

Figure 8 displays the energy profiles calculated employing ProSA web server for the initial and final models. The energy values higher than 0 of the initial model followed by same values lower than 0 for the final model indicate that the MD simulation was essential to stabilize the 3D structure.

## Figures and Tables

**Table 1:** Effect of inhibitors on enzymatic activity of SP.

Inhibitors	% inhibition of		
	S-2238	S-2222	S-2303
Benzamidine	62 ± 4	61 ± 2	47 ± 1
PMSF	55 ± 3	52 ± 3	45 ± 3
EDTA	7.5 ± 1	15 ± 2	5 ± 2
O-Phe	3.9 ± 2	3 ± 1	4.5 ± 2

The inhibitors in final concentration, benzamidine (15 mM), PMSF (2 mM), EDTA (20 mM), or O-Phe (0.3 mM) were preincubated with SP (20 *µ*g/mL) for 60 min at 37°C; then the reaction was initiated by adding chromogenic substrates (0.1 mM, final concentration). The reaction was monitored for 5 min, as described in the Section 2 and % inhibition was measured. 100% of the SP enzymatic activity was obtained in the absence of inhibitors for each substrate. Results are expressed as means ± SD of two individual experiments (*n* = 2).

**Table 2:** Comparison between thrombin and BpirSP-39 activities*.

Activities	Thrombin	BpirSP-39
Aggregation of platelet disaggregation	+	Not tested
Clot retraction	+	Not tested
Fibrinogen clotting	+	+
Factor XIII activation	+	+
Degradation of fibrinogen (*α* and *β*)	+	+
Hydrolysis of BAPNA	+	+
Inhibition by heparin	+	−
Inhibition by PMSF	+	+
Inhibition by citrate	+	−
Inhibition by EDTA	+	−

*Adapted from Niewiarowski et al., 1979 [61].

The presented data represents a summary of thrombin and BpirSP-39 activities.
