# Supplementary figures and images for: Isolation and Biochemical Characterization of a New Thrombin-Like Serine Protease from Bothrops pirajai Snake Venom
Source: Biomed Res Int. 2014 Feb 26;2014:595186. doi: 10.1155/2014/595186 (PMC3955695; doi:10.1155/2014/595186)

# Supplementary Material

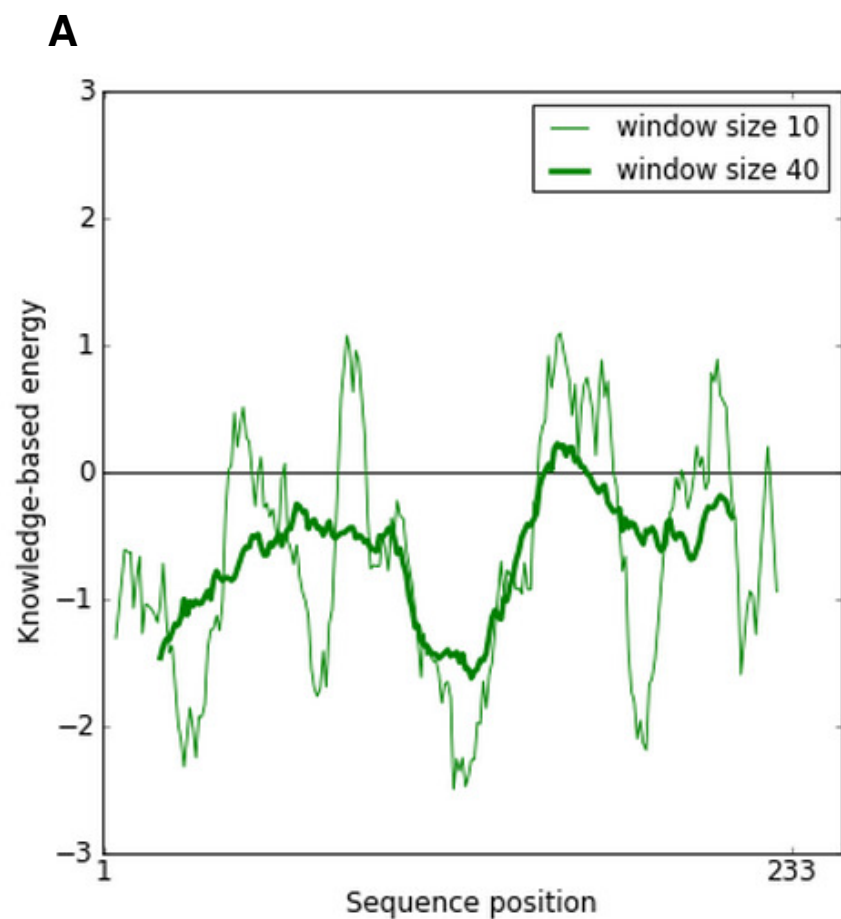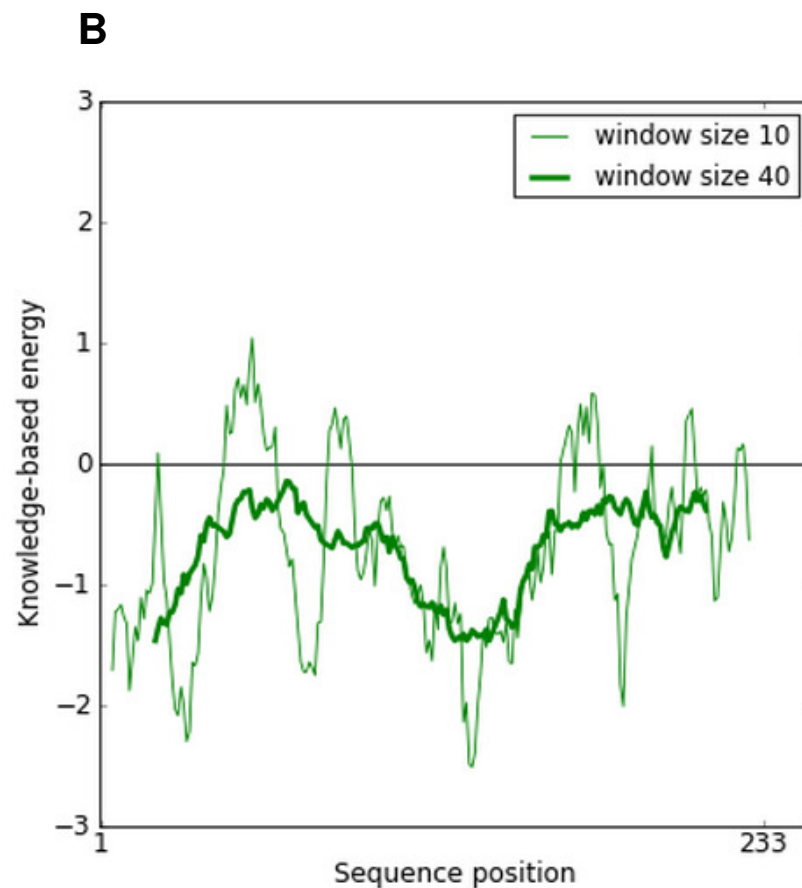

Supplement: Supplementary file 1 — Figure 8 displays the energy profiles calculated employing ProSA web server for the initial and final models. The energy values higher than 0 of the initial model followed by same values lower than 0 for the final model indicate that the MD simulation was essential to stabilize the 3D structure. [file 595186.f1.pdf]
